# Multi-Layer Beam Scanning Leaky Wave Antenna for Remote Vital Signs Detection at 60 GHz

**DOI:** 10.3390/s23084059

**Published:** 2023-04-17

**Authors:** Solomon Mingle, Despoina Kampouridou, Alexandros Feresidis

**Affiliations:** Department of Electronic, Electrical and Systems Engineering, University of Birmingham, Birmingham B15 2TT, UK

**Keywords:** 60 GHz band doppler radar sensors, beam scanning, contact monitoring device, leaky wave antenna, remote vital sign monitoring

## Abstract

A multi-layer beam-scanning leaky wave antenna (LWA) for remote vital sign monitoring (RVSM) at 60 GHz using a single-tone continuous-wave (CW) Doppler radar has been developed in a typical dynamic environment. The antenna’s components are: a partially reflecting surface (PRS), high-impedance surfaces (HISs), and a plain dielectric slab. A dipole antenna works as a source together with these elements to produce a gain of 24 dBi, a frequency beam scanning range of 30°, and precise remote vital sign monitoring (RVSM) up to 4 m across the operating frequency range (58–66 GHz). The antenna requirements for the DR are summarised in a typical dynamic scenario where a patient is to have continuous monitoring remotely, while sleeping. During the continuous health monitoring process, the patient has the freedom to move up to one meter away from the fixed sensor position.The proposed multi-layer LWA system was placed at a distance of 2 m and 4 m from the test subject to confirm the suitability of the developed antenna for dynamic RVSM applications. A proper setting of the operating frequency range (58 to 66 GHz) enabled the detection of both heart beats and respiration rates of the subject within a 30° angular range.

## 1. Introduction

Conventional methods of monitoring vital signs, such as an electrocardiogram (ECG), pulse oximetry, and capnography, require sensors to be attached directly to the patient’s body, which is either uncomfortable for the patient or not possible under certain circumstances [1,2,3,4,5,6,7,8]. Due to the fact that sensors do not require direct contact with the body, remote monitoring of vital signs with a Doppler radar is more convenient than conventional methods [8]. This makes remote monitoring of vital signs with a Doppler radar an attractive option. A Doppler radar is a non-contact technology that can detect small movements of the body caused by the heartbeat and breathing. This technology has the potential to revolutionize healthcare by providing continuous and non-invasive monitoring of vital signs, particularly for patients who require frequent monitoring or who are in critical condition. Remote vital sign monitoring (RVSM) with a Doppler radar has the potential to be used in a variety of disciplines, including general and specialised healthcare, emergency services, security, and defence. However, for RVSM to be useful in practice, further research needs to be conducted on the accuracy and reliability of the results in different settings and populations [1,2,3,4,5,6,7,8,9,10].

The main advantages of using millimetre-wave frequencies for RVSM are (i) higher detection sensitivity due to shorter signal wavelengths, (ii) smaller form factors for more compact devices, and (iii) the ability to transmit and receive signals more precisely without interference from the environment [4,9]. Conventional methods such as electrocardiography (ECG) and photoplethysmography (PPG) require physical contact with the subject, which can be uncomfortable and can interfere with normal activity. Additionally, ECG and PPG provide measurements at a single point in time, while millimetre-wave technology can provide continuous monitoring over an extended period. Furthermore, ECG and PPG are often limited in their ability to penetrate through clothing and tissue, which can limit their accuracy in certain situations.

**Figure 1:**
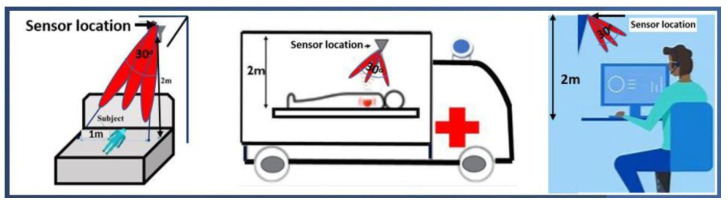
Examples of RVSM applications.

The 60 GHz frequency band (57–66 GHz) has attracted RVSM interest due to its licence-free nature and its widespread use for several different wireless services. Antennas that operate in this band are particularly challenging to design and fabricate. RVSM measurements require the focus of the antenna beams, as well as enough gain to compensate for path loss in order to achieve high precision. Moreover, antenna beam steering capability is necessary to continuously monitor health with a fixed position sensor over a long period of time, especially when the person may move in practical scenarios, such as during sleep, inside a room, during transport in an ambulance, and at work [11,12,13]. Some examples of beam-steered antenna applications for RVSM are depicted in Figure 1.

Recent publications [4,8,9] discuss the design of mm-wave antennas for health monitoring with a Doppler radar. However, these are fixed beam antennas, and their gain does not exceed 20 dBi. Recent reports have shown that digital beamforming antenna systems operating in the mm-waveband can be used in a variety of industrial and automotive applications. In RVSM sensors, spatial arrays, compressive sensing, route sharing, and MIMO antennas have high power requirements, which limits their application. Furthermore, their hardware is complicated and expensive. The development of beam steering antennas for use in health monitoring on mm-waves has received a limited number of papers to the best of our knowledge. According to [11,13,14], this is likely the result of the limited frequency of electronic components available today.

Leaky wave antennas (LWAs) are a special family of antennas that are able to steer their beam with frequency [15]. They provide a number of advantages that makes them very suitable for RVSM application. These advantages include beam steering with frequency, which allows the antenna beam to be directed towards the subject of interest without any mechanical rotation; high directivity and bandwidth, which improves the signal-to-noise ratio and increases the range of the system; low profile, which makes such antennas suitable for use in compact and portable monitoring systems.

Our study presents a multi-layer LWA used for remote vital signs detection in a typical situation in which a patient is lying on the bed and has some random movements during sleep. The non-contact detection of vital signs can be achieved using antenna beam characteristics that provide high gain (58–66 GHz) and wide bandwidth performance. A measured antenna bandwidth of 8 GHz and a maximum gain of 24 dBi are measured across the operating band. The proposed antennas are tested experimentally to validate their predicted detection coverage. This antenna design measures the respiratory rate (BR) and the heart rate (HR) from a distance of up to 4 m from the body of the person at five different radiation angles between 6° and 36°. A very good agreement between the measured results and the predicted and simulated results has been achieved.

This work is an improvement from the antennas already published in [16,17]. The reported antenna bandwidth, maximum gain, and beam scanning range in [16] are 3.78 GHz, 20.35 dBi, and 12°, respectively, compared to 16 GHz, 24 dBi, and 30° in this work. The dielectric image line antenna of [17] has a maximum gain of 19 dBi as compared to 24dBi gain of the proposed multi-layer LWA. Due to this high gain, the multiplayer LWA in this work has more advantages in terms of distance applications, such as radar, and many IoT applications.

This paper is organised as follows: The antenna design is described in Section 2, antenna measurements and discussions are presented in Section 3, health monitoring measurements are discussed in Section 4, and this work is brought to a conclusion in Section 5.

## 2. Antenna Design Methods

### 2.1. Unit Cell Design

To analyse the proposed design, we begin by applying ray optics in order to measure multiple reflections between partial reflective surfaces (PRSs) and high impedance surfaces (HISs) on the ground plane [18,19]. The PRS and HIS reflection characteristics are related to antenna gain, bandwidth, and radiation patterns. The same periodicity *P* is selected for the HIS and PRS unit cells. The reflectance properties of PRS and HIS unit cells are determined at normal incidence. More details about this analysis technique and the relevant equations can be found in [18,19,20,21,22].

**Figure 2:**
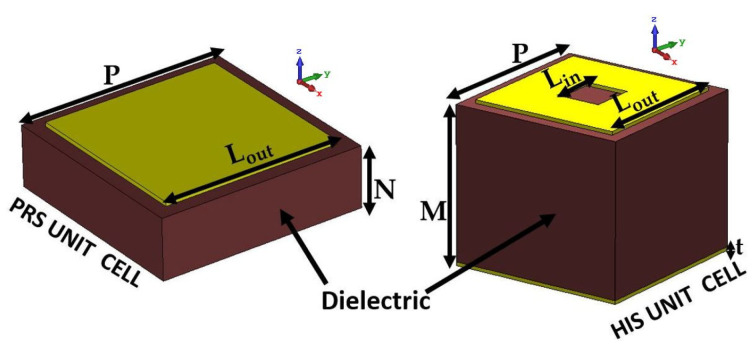
PRS and HIS unit cell structure where $L_{out}=1.50$ mm, $L_{in}=0.60$ mm and periodicity *P* = 1.75 mm.

Both PRSs and HISs are metasurface arrays made up of square patch elements and square ring elements, respectively, with a ground plane etched on a planar printed circuit board. Figure 2 depicts the structures of both unit cells. The metasurface elements are printed on a Rogers RT/Duroid5880 substrate with a dielectric constant of $\epsilon_{r}=2.2$ and a tangent loss of 0.0019.

**Figure 3:**
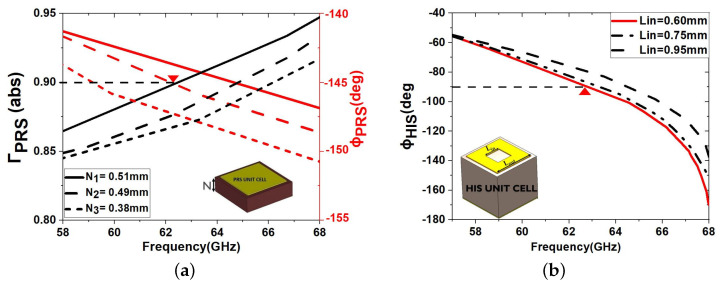
(**a**) Magnitude and phase reflection coefficient of the PRS unit cell. (**b**) Phase reflection coefficient of the HIS unit cell with respect to $L_{in}$ with $L_{out}$ = 1.50 mm.

The design process of the PRS and HIS unit cells is depicted in Figure 3 with respect to important parameters. For both unit cells, the copper cladding thickness is 0.035 mm. The substrate thickness for PRS is selected at N=0.508 mm so that a highly reflective response is achieved, which is necessary for a high gain antenna (Figure 3a). The top patch is square in order to maximize the reflection coefficient magnitude and symmetry [19,22,23]. The HIS unit cell of M=1.57 mm (Figure 3b) uses a square metallic ring element with an inner loop, optimised to achieve a phase change with frequency [18]. The parametric study of Figure 3b for a selected dimension $L_{out}=1.5$ mm shows that a rapid phase change is achieved with a selection of $L_{in}=0.6$ mm.

Periodic boundary conditions were utilised in CST Microwave Studio^TM^ along the *x* and *y* axis for both unit cell simulations. Open boundaries were established along the incident wave’s +z-axis in accordance with the design process first detailed in [21] to extract the reflection coefficients and optimize the dimensions.

Figure 3a shows the simulated reflection coefficient results of the PRS unit cell, exhibiting an absolute magnitude |Γ|=0.9 and reflection phase $\phi_{PRS}$ response = 145° at the frequency of 62.5 GHz, respectively, with slow variation in frequency. Figure 3b depicts the simulated reflection coefficient of $S_{11}$ results of the HIS unit cell, which demonstrate a phase response of −95° at 62.5 GHz. This conforms to the pattern of reflection that is expected from free-standing frequency selective surfaces (FSSs). From the simulated values at 62.5 GHz, the ray tracing method predicts an antenna bandwidth (BW) and a gain of approximately 8 GHz and 24 dBi, respectively, for PRS layers with an finite thickness when fed through by a dipole.

### 2.2. Printed Dipole Antenna Design

**Figure 4:**
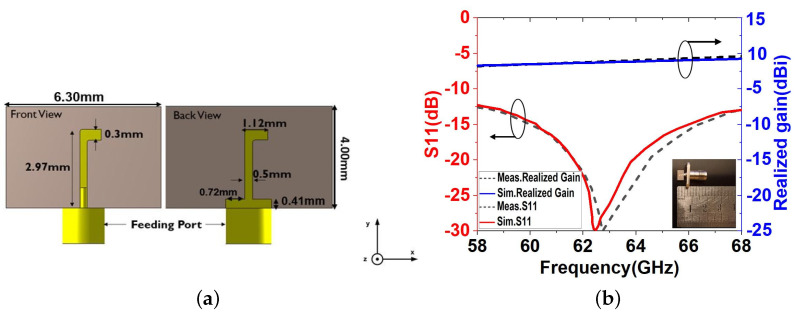
The feeding PDA structure. (**a**) dimensions in front view and back view; (**b**) simulated and measured $S_{11}$ and realised gain.

In this section, a printed dipole antenna (PDA) is designed, which will serve as a primary feeding for the complete leaky wave antenna structure. A design model of this feeding dipole antenna can be seen in Figure 4a. Its dimensions are optimised for best performance in the 60 GHz frequency range. The printed dipole antenna (PDA) is etched on RT/Duroid 5880 with a dielectric constant of $\epsilon_{r}$ = 2.2 and a thickness of 0.13 mm. A reflector is placed on the backside of the printed dipole antenna (PDA) at an optimised distance of 0.41 mm to improve the functionality of the LWA in the xy plane [24]. Figure 4b shows that the simulation and measured results of $S_{11}$ and realised gain are in good agreement.

### 2.3. Multi-Layer LWA Design

**Figure 5:**
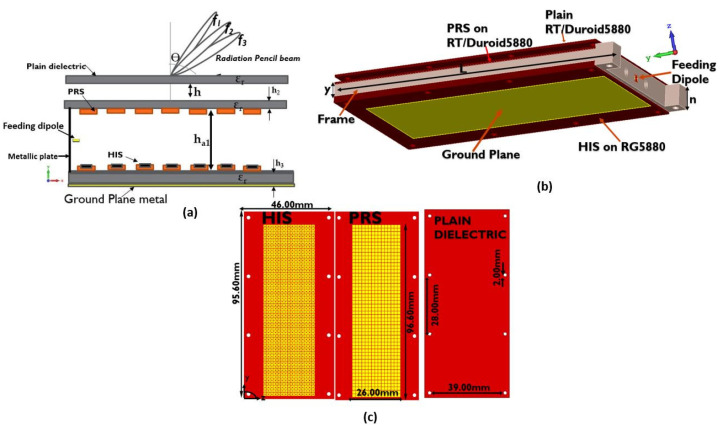
Proposed antenna structure: (**a**) the geometry of LWA; (**b**) view of the LWA along the xy plane; (**c**) 3D view of the LWA with a frame where *y* = 5 mm, *n* = 5.2 mm, and *L* = 102 mm.

The full proposed multi-layer LWA design is shown in Figure 5b. Both the partially reflective surface (PRS) and the high impedance surfaces (HISs) are formed by arrays of 50×15 elements. This size of the antenna was selected as a compromise between a relatively compact size, a satisfactory high gain, and the infinite size assumption of leaky wave theory. The rectangular shape of the antenna was selected in order to achieve a highly directive beam at the E-plane. A fed dipole (Figure 5a) is positioned in the cavity between PRS and HIS towards one end of the antenna. On the same side, there is a vertical metallic wall where the antenna is terminated (Figure 5b). A 3D view of the antenna elements is depicted in Figure 5c.

The height of the cavity between the PRS and the HIS ground plane, which is represented by the symbol $h_{a1}$ is calculated to be 2.90 mm. Following the ray optic method introduced in [25], the pointing angle of the radiation pattern “*P*” can be predicted as:

(1) $$P=\frac{1-\Gamma^{2}}{1+\Gamma^{2}-cos\left(\phi_{PRS}-\phi_{HIS}+\frac{4\pi }{\lambda_{0}}h_{a1}cos\theta \right)}F\left(\theta \right)2$$

where F(θ) equal to 1, Γ and $\phi_{PRS}$ represent the magnitude and phase of reflection coefficients of PRS, and λ is the operating wavelength.

**Figure 6:**
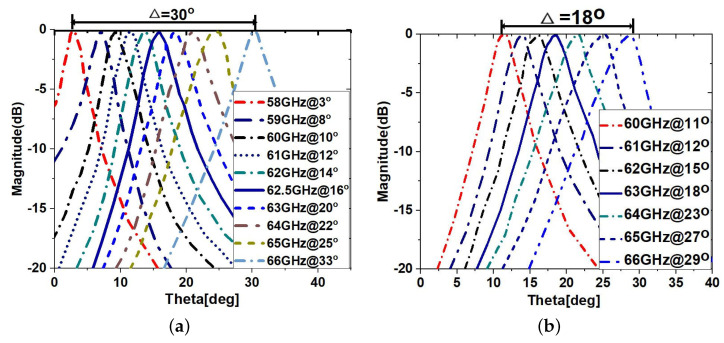
Theoretically estimated beam scanning range of the LWA from 58 to 66 GHz for a fixed cavity height of (**a**) $h_{a1}$ = 2.90 mm with HIS and (**b**) $h_{a1}$ = 3.32 mm without HIS.

**Figure 7:**
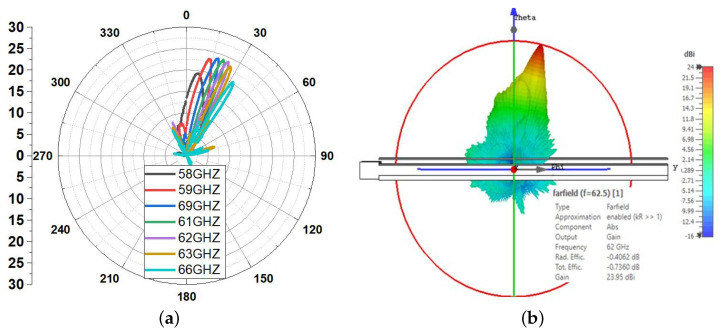
LWA simulation results: (**a**) 2-D FRPs in *E*-plane at various frequencies within the operating band; (**b**) 3-D FRP of the proposed LWA at 62.5 GHz.

The phase reflection coefficients of PRS and HIS as a function of frequencies are used to demonstrate LWA beam scanning. These coefficients are derived using Equation (1), which produces a wide scanning angle of 30°, as shown in Figure 6a. It was found that when HIS is replaced by a ground plane, the beam scanning reduces drastically to 18° with few frequencies, as shown in Figure 6b. This is because the ground plane is not sensitive enough to produce high phase reflection coefficients compared to HIS arrays. The cavity height $h_{a1}$ and maximum gain calculation can be found in [20]. The far-field radiation patterns (FRPs) of the proposed antenna design are shown in Figure 7a for a cavity height of 2.90 mm. In particular, Figure 7a depicts the 2-D FRP in the *E*-plane at various frequencies within the operational range, and Figure 7b depicts the 3-D FRP at 62.5 GHz.

To prove the design concept, three additional variations of our proposed multi-layer LWA were considered, which have been demonstrated through simulations in CST Microwave Studio. Our proposed multi-layer LWA consists of a HIS-PRS cavity and a dielectric on top (as in Figure 5). Three variations of this model are compared: the proposed HIS-PRS LWA of Figure 5 without the dielectric layer on top, the LWA of Figure 5 with a mere ground plane instead of a HIS, and the proposed LWA with a mere ground plane and without the top dielectric layer.

**Figure 8:**
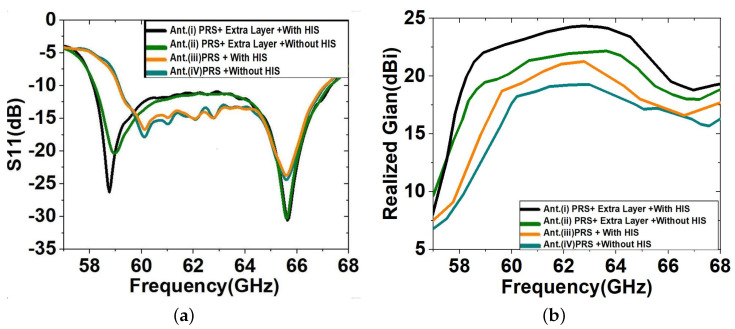
Simulated LWA models (**a**) $S_{11}$ and (**b**) realised gain for four different LWA configurations.

The $S_{11}$ parameters for the gain comparison for these four cases are depicted in Figure 8. The $S_{11}$ (Figure 8a) is well below −10 dB for all four LWA models for cavity heights $h_{a1}$ = 2.90 mm (antenna with HIS) and $h_{a1}$ = 3.32 mm (antenna without HIS), and the realised gain remains above 19 dBi for the frequency range of 58 to 67 GHz. It is furthermore evident from Figure 8 that the proposed multi-layer LWA impedance matching, gain, and $S_{11}$ bandwidth are significantly improved (24.3 dBi and 8 dB) by adding an extra plain dielectric slab over the PRS array with a beam scanning range of about 30° and a scanning loss of 3 dB. The proposed multi-layer LWA with HIS and dielectric exhibits a narrower half-power beamwidth (HPBW) compared to the other configurations, spanning from 9° at 58 GHz to 6° at 66.7 GHz. Additionally, the sidelobe level of the proposed multi-layer LWA remains below −10 dB in all the frequency bands. The antenna’s simulated results are closely related to their previously calculated expected results. The aforementioned results are summarised in Table 1.

## 3. Multi-Layer LWA Measured Results and Discussion

**Figure 9:**
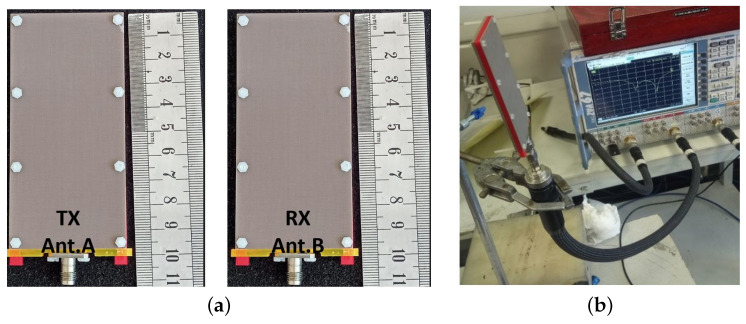
(**a**) Fabricated LWA prototype with external connections (ant. and ant. B) (**b**) $S_{11}$ test setup.

The entire antenna design, including the coaxial feed, is encased in a low-loss polytetrafluoroethylene (PTFE) so that it can be placed in the most realistic environment possible and measurements can be made. An external connection is made using a 1.85 mm flanged launcher and a GB185 glass bead. The antenna layers were fabricated using a typical low-cost PCB fabrication method. The measurement configuration for the multi-layer LWA’s $S_{1}1$ prototype photo is shown in Figure 9, along with two prototypes of the proposed LWA (ant. A and B) for RVSM application.

The measurement of the antenna was carried out in an anechoic chamber. The gain-comparison method was used to determine the realised gain of our proposed multi-layer, leaky wave antenna. This method calls for two antennas: one that serves as a receive antenna and has a known gain (in our case, a horn antenna) and another that serves as a transmitter antenna whose gain is unknown (our proposed multi-layer LWA). The antennas were placed at a far-field distance of 1 m. The antenna gain was next calculated using Friis’ equation. 

**Figure 10:**
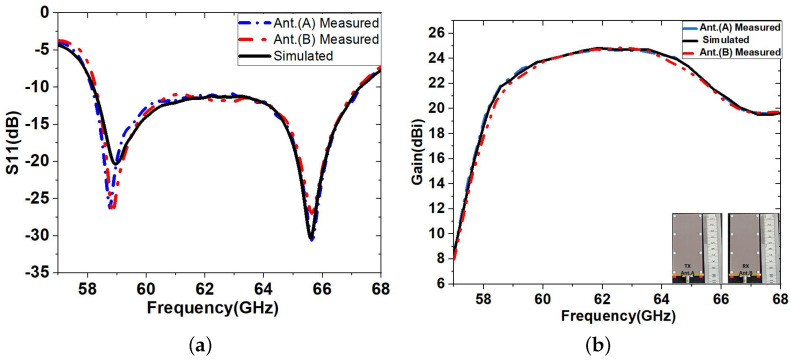
Measured LWA results compared to simulated in (**a**) $S_{11}$ and (**b**) Realised gain.

A rotatable base connected to the computer for data acquisition was used to measure the patterns of antenna radiation. The antenna is rotated through 0°, 10°, 20°, 30°, up to 360° and then returned to the starting position after calibrating the VNA to 0 dB insertion loss. The information is then gathered and kept on file to forecast the signal strength. Figure 10 compares the simulated and measured results of the $S_{11}$ and the gain of the proposed multi-layer LWA for RVSM. The measured values for the $S_{11}$ bandwidth and the maximum gain of ant. A is 7.89 GHz and 23.95 dBi, respectively, which are slightly higher than the simulation values (6.8 GHz and 23.5 dBi). Over the key frequencies of interest ranging from 58 to 66 GHz, the *S*-parameter stays lower than −10 dB. In addition, the beam scanning ranges of each of the tested antennas are the same, with a scanning range of 30°. The small discrepancies between simulations and measurements are attributed to errors during the fabrication process, which affect the performance of the antenna at mm-wave frequencies.

**Figure 11:**
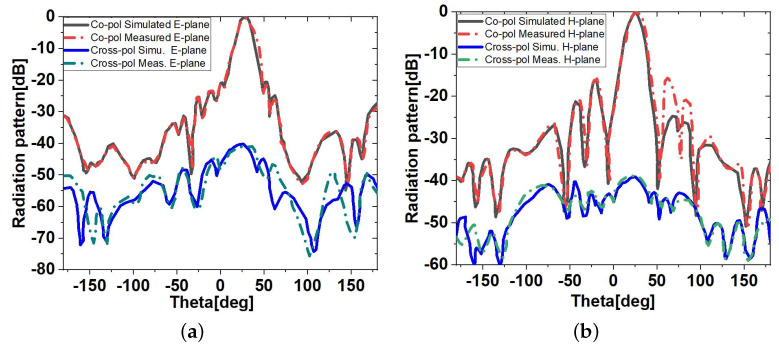
Measured and simulated copolar and crosspolar (**a**) *E*-plane and (**b**) *H*-plane radiation patterns at 62.5 GHz.

**Figure 12:**
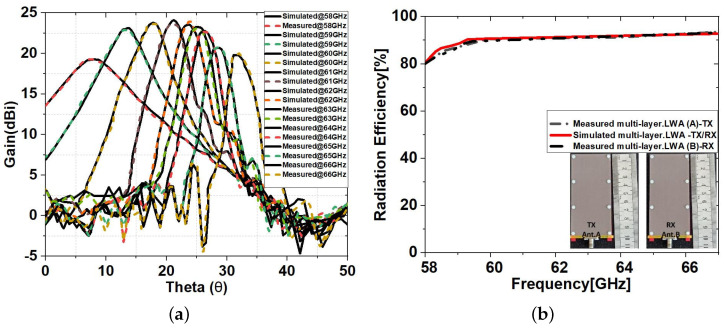
The measured and simulated (**a**) *E*-plane radiation patterns of the multi-layer LWA and (**b**) radiation efficiency.

To validate the design concept, the measured far-field radiation patterns of the co-polar and cross-polar in the *E*-plane and *H*-plane radiation patterns at 62.5 GHz are presented in Figure 11. Manufacturing and radiation measurement tolerances, such as link losses, lateral reflections, and antenna misalignment, may be to blame for any differences between the simulated and observed $S_{11}$ and FRPs. A complete comparison between simulated and measured *E*-plane radiation patterns at different frequencies is given in Figure 12a. They show excellent agreement between simulations and measurements.

Both the measured and simulated efficiency of antennas A and B remained at approximately 95% for the frequency range above 60 GHz (Figure 12b). For this measurement, first, a current was run through the antenna terminals, and then the strength of the electromagnetic field that went out into space was measured. In the next section, the measured antenna characteristics will be used for the RVSM experiment.

## 4. RVSM Measurements with Doppler Radar

In this experiment, we focus only on heart rate and respiration rate measured on the chest. Our proposed leaky wave antenna with a beam scanning range of 30° is sufficient for this type of experiment. However, it should be noted that to scan other parts of the body with this method, more complex systems should be used that reach beyond the scope of this paper.

**Figure 13:**
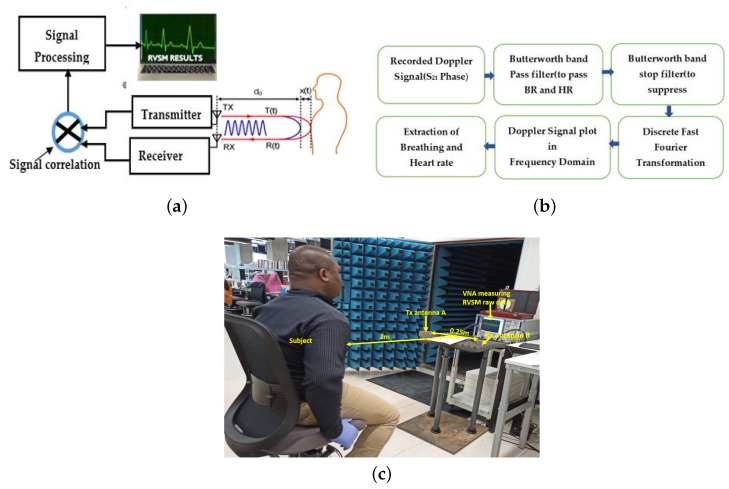
Demonstration of the RVSM experiment setup measurements.

The proposed RVSM process is shown in a block diagram in Figure 13a. The transmitting antenna (Tx ) transmits a one-tone electromagnetic (EM), continuous wave (CW signal) in RVSM using a DR technique for a predetermined period of time. After the electromagnetic signal is scattered on the chest of a person standing at a determined distance in front of the antennas, it is simultaneously picked up by a receiving antenna (Rx). The quasi-periodic oscillations in the chest caused by breathing and heartbeats during the designated time phase modulate the received signal. The received phase-modulated signal and the transmitted signal are correlated for demodulation. The time domain (TD) information for the roughly recorded baseband signal at the Rx is provided below [4]:

(2) $$R\left(t\right)=cos[\theta \left(t\right)+\frac{4\pi x_{b}\left(t\right)}{\lambda }+\frac{4\pi x_{h}\left(t\right)}{\lambda }]$$

where θ(t) represents the total phase shift brought on by the signal path (*d*), reflected signals from the environment and the subject, and residual phase noise. The $x_{b}\left(t\right)$ and $x_{h}\left(t\right)$ are the vibrational shifts of the chest that are, respectively, represented by the breathing and heartbeat, where λ is the operating wavelength. These shifts occur when the chest is subjected to vibration. Due to the periodic nature of $x_{b}\left(t\right)$ and $x_{h}\left(t\right)$, an approximation of them may be made as follows: $x_{b}\left(t\right)=m_{b}\;sin\left(2\pi \;f_{b}\;t\right)$ and $x_{h}\left(t\right)=m_{h}\;sin\left(2\pi \;f_{h}\;t\right)$, where $m_{b}$ and $m_{h}$ are the displacement amplitudes of the chest vibration. The recorded demodulated received signal can be expanded in a Fourier series in the manner described below and thus translated into the Frequency Domain (FD) [26]:

(3) $$\begin{array}{c} R\left(t\right)=\sum_{i=-\infty }^{\infty }\sum_{j=-\infty }^{\infty }J_{j}\left[\frac{4\pi m_{b}}{\lambda }\right]J_{i}\left[\frac{4\pi m_{h}}{\lambda }\right]\times \\ cos(j2\pi \;f_{b}\;t+i2\pi \;f_{h}\;t+\theta ) \end{array}$$

where J(X) is the argument X representing a Bessel function of the first order. Equation (3) above contains the required BR and HR details, in addition to noise distortions and vibrations from the surroundings. After that, the appropriate digital filters are applied to Equation (3) in order to separate the essential BR and HR signals from the background noise as well as the harmonics that are not required. Figure 13b depicts the whole of digital signal post-processing. Figure 13c shows the experimental setup for the RVSM. The vector network analyser (VNA) with a maximum frequency limit of 67 GHz was used as the transceiver (TRx), which has the proposed Tx and Rx antenna modules connected to it for our RVSM measurements. An extensive series of experiments was carried out to verify the region that the beam-scanning antennas predicted for detecting vital signs while stationary.

To accomplish this, the VNA is first calibrated to send and receive electromagnetic waves with a transmit power of 0 dBm and 201 sampling points in the frequency range between 58 and 66 GHz. As shown in Figure 13c, the test subject sits at a certain radial distance and angular position in front of the antennas. A CW sweep with a tone for 60 s is performed. In a short time, the Tx antenna on port 1 of the VNA sends out a signal, and the Rx antenna on port 2 picks up the reflected signal that the subject sends back. The phase of the received signal is demodulated and recorded by the VNA for 60 s in an $S_{21}$-phase format. The next step is to extract the needed BR and HR data from the recorded $S_{21}$ phase data through a signal processing programme in MATLAB. The main parts of the signal processing program are bandpass filters that allow BR and HR frequencies, discrete fast Fourier transform (DFFT), and notch filters (10th order Butterworth digital filters) that reduce noise, read out data, and display the heart rate and breathing rate. The values of BR and HR was compared with a hospital blood-pressure monitor for heart rate and manually counted average values of BR to make sure the measurements are accurate. For this reason, the experiment recorded the pulse values of BR and HR five times each and compared their average values with the values of BR and HR obtained by the DR system. For this reason, electrocardiograms (ECGs) are not used to compare waveforms. The reason for this is that, in the presented beam scanning DR to support the possibility of RVSM detection in connection with the suggested antenna beam steering, we are only interested in the average BR and HR (1/min) results in FD (and not in the BR and HR waveforms in time).

Fourier transform-based DR signalling has a RVSM acquisition time and BR/HR resolution trade-off, which must be strictly balanced. For instance, a signal recorded for 60 s will result in a frequency resolution of 1 pulse/s. Likewise, a recorded signal time of 30 s will reduce the BR/HR resolution to 0.5 pulses/s. Signal processing uses time windows. The DR signal is continuously captured in time windowing, but the signal processing is applied to a predetermined shorter time period (e.g., 60 s for a pulse resolution of 1/min), and the findings are updated after a shorter time interval (e.g., every 5 s) [27]. High-resolution RVSM results may be averaged across time using Fourier transform-based signal processing. Other complex signal-processing techniques can be seen in [14].

**Figure 14:**
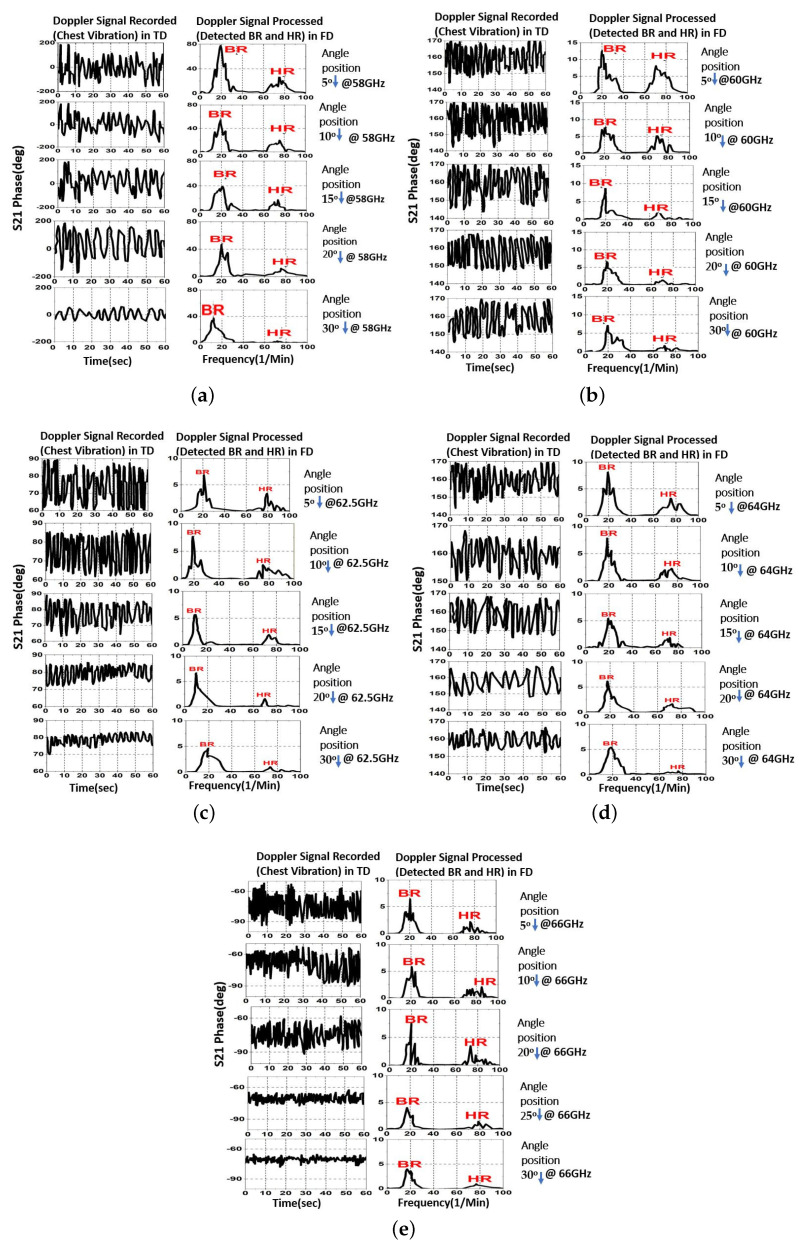
Measured RVSM results from 2 m distance at (**a**) 58 GHz, (**b**) 60 GHz, (**c**) 62.5 GHz, (**d**) 64 GHz, and (**e**) 66 GHz.

In this experiment, the target is 2 m away from the transmitter and the receiver antennas at 5°, 10°, 15°, 20°, and 30°. For each angular point, the demodulated Doppler signal in TD, which contains the $S_{2}1$ phase data, is recorded at 58 GHz. The results are replicated for frequencies of 60 GHz, 62.5 GHz, 64 GHz, and 66 GHz. As a result, we are able to collect five sets of $S_{2}1$ phase data at the five specified angular locations for 58 GHz, 60 GHz, 62.5 GHz, 64 GHz, and 65 GHz, as shown in Figure 14 and Table 2. On the left of each subplot is the Doppler signal that was recorded in TD, and on the right is the corresponding processed signal in FD. It can be seen that the Doppler signal with the biggest amplitude compared to the level of background noise was picked up at a wider angle when the operating frequencies were higher. A good illustration of this is the modest peak HR, which is more easily detected by noise.

The estimated BR signal amplitude is seen in the first peak at a frequency of about 19 (1/min), while the predicted HR signal amplitude is seen in the second peak at a frequency of about 74 (1/min). At angles of 5° and 15°, the observed BR and HR signals exhibited amplitudes that were considerably larger than the noise values for the locations under examination. The results confirm predictions that the antenna radiation beam at 62.5 GHz is centred at roughly 25° and has an HPBW of greater than 12° (see Figure 11a and Table 2). It is also evident that the largest and clearest HR peak at 58 GHz is at 5° and 15°, whereas at 62.5 GHz and 64 GHz, it is at 5°, 15°, 25°, and 30°, respectively.

The HR peak is still visible at 66 GHz, and it is greater than the surrounding noise at 30° (See Figure 14e). It can be concluded that the RVSM detection response of the proposed antenna is suitable for a distance of 2 m at an angular range of 5° to 30°, which corresponds to an angular separation of 0.9 m when the operating frequency oscillates between 58 GHz and 66 GHz. Due to the antenna gain and transmit power, the angular range for RVSM detection can be increased beyond 0.9 m if the antennas are placed far away from the object [28].

**Figure 15:**
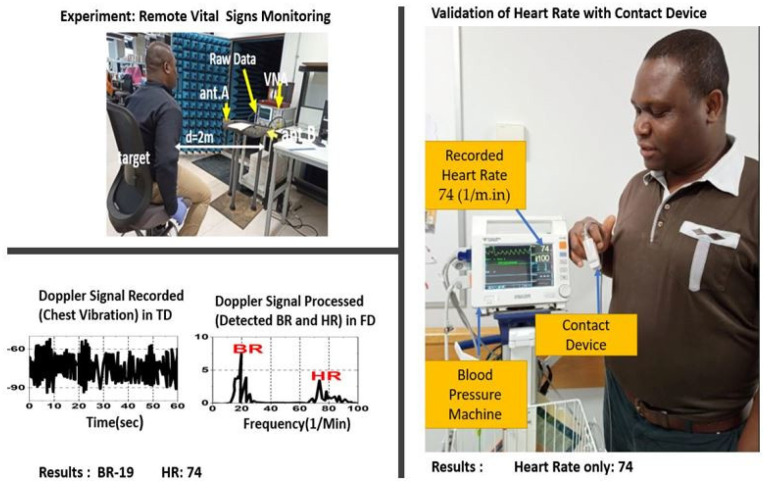
Validation of RVSM using (LHS) non-contact device (RHS) traditional blood pressure machine.

A hospital blood pressure monitor that also records patients’ heart rates on a daily basis was used to validate the results on the same individual, as shown in Figure 15. The BR and HR results from the experiment are compared with the results from the contact device, and it can be seen that both sets of results are in good agreement. This experiment, therefore, demonstrates that the proposed antenna can be used to measure both heart rate and breathing for longer distances (2–4 m) from the subject to provide more than 2 m of angular coverage.

**Figure 16:**
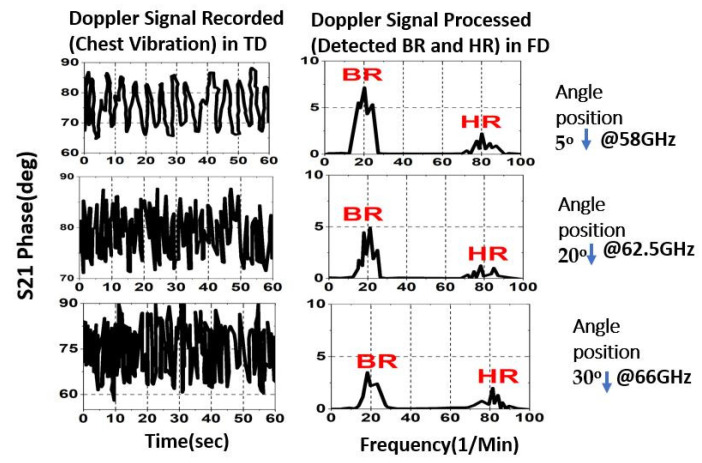
Measured RVSM results from a 4 m distance at 58 GHz, 62.5 GHz, and 66 GHz.

In order to demonstrate the antennas’ range at a radial distance of 4 m, the experiment was repeated multiple times with the same subject, who sat at varying angles away from the antennas each time and had their vital signs recorded. The purpose of this was to validate the results and compare them to those obtained from a range of 2 m. The other experimental parameters are kept as previously described. Three RVSM measurements were taken at the best frequencies and beam angles for maximum detect ability, according to Figure 16: 58 GHz for 5°, 62.5 GHz for 25°, and 66 GHz for 30°.

The proposed multi-layer LWA design with Doppler radar technology is well-suited for use as a non-contact wireless sensor for health monitoring. As the experiment has shown, the sensor can detect both breathing rate (BR) and heart rate (HR) accurately and reliably at a radial distance of up to 4 metres. The non-contact nature of the sensor means that it can be used in a variety of scenarios, including when a patient is travelling in an ambulance or when they are sleeping or in occupational settings, where workers’ health can be monitored without the need for physical contact (see Figure 1). Additionally, the sensor’s ability to measure breathing rate and heart rate parameters within an angular range of 5° to 30° makes it a versatile tool which can be used in different settings. The proposed LWA design’s potential applications are not limited to healthcare; however, the sensor’s ability to detect and monitor physiological parameters from a distance could also be useful in sports, where athletes’ performance and recovery can be monitored remotely.

Table 3 compares this work with others from relative recent literature with RVSM applications. Overall, the major contribution of our proposed LWA is the scanning distance of up to 4 metres from the human body with an improved accuracy of 99.3%, which is a significant advancement compared to other antennas and systems that have been used for RVSM. Additionally, our proposed antenna operates with an enhanced realised gain of 24.3 dBi and radiation efficiency of 95.5% compared to relevant work from the literature. The achieved bandwidth of our work is more limited compared to [17], which is due to the well-known trade-off between gain and beam aperture, which explains the more limited range of our antenna compared to [17]. However, the obtained realised gain of our proposed design makes it suitable not only for RVSM but for a number of other applications as well, such as 5G communications and IoT.

## 5. Conclusions

The proposed multi-layer leaky wave antenna is a highly promising technology due to its numerous advantages. It operates in the 60 GHz band, which is ideal for applications requiring high data rates, low interference, and short-high-range communication. Additionally, the antenna has a high gain and enough beam-steering range for RVSM (Remote Vital Sign Monitoring) applications, which is essential for accurately tracking the movements of a person’s body. It is manufactured using low-cost PCB (Printed Circuit Board) fabrication methods, which makes it a highly attractive option for widespread adoption. Additionally, the design has been tested and characterised through two prototypes, and good agreement has been observed between the simulations and measurements. The experiment conducted to test the antennas for respiration and heartbeat detection showed that the proposed antenna is highly effective. The antenna was tested at radial distances of 2–4 m and angular positions of 5° to 30° on the subject’s body, with successful results. This indicates that the antenna is capable of accurately detecting BR and HR parameters from a distance, making it ideal for use in healthcare and other fields. The design method could be used to make low-cost antennas with high gain that can also work at higher mm-wave frequencies and have a wider range of radiation. 

## Figures and Tables

**Table 1:** Comparison of simulated LWA results at a resonant frequency of 62.5 GHz.

Antenna Type	Operating Band $S_{11}<-10$ dB	Realised Gain (dBi)	Beam Angles (deg)	HPBW (deg)
**with HIS**	58–67 GHz	22	10°–33°	12°–5°
**without HIS**	60–66.6 GHz	19.8	13°–33°	14°–6°
***with HIS*** ***and dielectric***	*58–66.7 GHz*	*24.3*	*4°–34°*	*9°–6°*
**without HIS** ** and dielectric**	60–66.7 GHz	18	12°–33°	16°–7°

**Table 2:** Measured BR and HR with HIS-PRS LWA operation results at angles of 5°, 10°, 15°, 20°, and 30° for each frequency.

58 GHz	60 GHz	62.5 GHz	64 GHz	66 GHz	Manually Counted BR and HR Range with Hospital Machine		Max. Error
BR-HR (1/min)	BR-HR (1/min)	BR-HR (1/min)	BR-HR (1/min)	BR-HR (1/min)	BR (1/min)	HR (1/min)	BR-HR (1/min)
20–79	19–78	20–78	20–78	21–80	19–21 (avg. 20)	78–80 (avg. 79)	5–2%

**Table 3:** Comparison of the proposed LWA with literature.

	[10]	[14]	[16]	[17]	This Work
**Frequency (GHz)**	60	24	62–65	50–66	**58–66**
**Radiation efficiency (%)**	94	-	95	83.2	**95.5**
**Scanning range (°)**	0	90	12	70	**30**
**Gain (dBi)**	24	-	20.1	19.3	**24.3**
**Bandwidth (%)**	12	-	3.38	16	**8**
**RVSM distance (m)**	1	3.4	3	1.5	**4**
**RVSM accuracy (%)**	98.6	99.12	90.8	95	**99.3**

## Data Availability

The data that support the findings of this study are available from the corresponding author upon reasonable request.
